# Diagnosis of latent tuberculosis infection in healthy young adults in a country with high tuberculosis burden and BCG vaccination at birth

**DOI:** 10.1186/1756-0500-5-415

**Published:** 2012-08-07

**Authors:** Alemnew F Dagnew, Jemal Hussein, Markos Abebe, Martha Zewdie, Adane Mihret, Ahmed Bedru, Menberework Chanyalew, Lawrence Yamuah, Girmay Medhin, Peter Bang, T Mark Doherty, Asrat Hailu, Abraham Aseffa

**Affiliations:** 1 Armauer Hansen Research Institute, Addis Ababa, Ethiopia; 2 Department of Microbiology, Immunology and Parasitology, Faculty of Medicine, College of Health Sciences, Addis Ababa University, Addis Ababa, Ethiopia; 3 Department of Microbiology, Immunology and Parasitology, College of Health Sciences, Mekelle University, Mekelle, Ethiopia; 4 Aklilu Lemma Institute of Pathobiology, Addis Ababa University, Addis Ababa, Ethiopia; 5 Statens Serum Institut, Copenhagen, Denmark; 6 Current address: GSK, Brøndby, Denmark; 7 Current address: Global Development, Novartis Vaccines and Diagnostics, Via Fiorentina 1, Siena, 53100, Italy

**Keywords:** Tuberculosis, Latent, BCG, Tuberculin skin test, Interferon-γ release assay, Ethiopia

## Abstract

**Background:**

One third of the world’s population is thought to have latent tuberculosis infection (LTBI) with the potential for subsequent reactivation of disease. To better characterize this important population, studies comparing Tuberculin Skin Test (TST) and the new interferon-γ release assays including QuantiFERON®-TB Gold In-Tube (QFT-GIT) have been conducted in different parts of the world, but most of these have been in countries with a low incidence of tuberculosis (TB). The aim of this study was therefore to evaluate the use of QFT-GIT assay as compared with TST in the diagnosis of LTBI in Ethiopia, a country with a high burden of TB and routine BCG vaccination at birth.

**Methods:**

Healthy medical and paramedical male students at the Faculty of Medicine, Addis Ababa University, Ethiopia were enrolled into the study from December 2008 to February 2009. The TST and QFTG-IT assay were performed using standard methods.

**Results:**

The mean age of the study participants was 20.9 years. From a total of 107 study participants, 46.7% (95%CI: 37.0% to 56.6%) had a positive TST result (TST≥10 mm), 43.9% (95%CI: 34.3% to 53.9%) had a positive QFT-GIT assay result and 44.9% (95%CI: 35.2% to 54.8%) had BCG scar. There was strong agreement between TST (TST ≥10mm) and QFT-GIT assay (Kappa = 0.83, *p* value = 0.000).

**Conclusion:**

The TST and QFT-GIT assay show similar efficacy for the diagnosis of LTBI in healthy young adults residing in Ethiopia, a country with high TB incidence.

## Background

Tuberculosis (TB) is one of the leading causes of morbidity due to infectious disease, and its ability to establish latent infection compounds the difficulty of controlling the spread of infection. The primary cause of TB in humans is *Mycobacterium tuberculosis* (MTB) which together with *M. bovisM. bovis* Bacille Calmette-Guérin (BCG), *M. africanum, M. microti*, and *M. canetti* makes up the *M. tuberculosis* complex (MTBC) [1]. Exposure to a person with active TB, leading to infection, can result in one of the three outcomes [2]. The innate immune system can rapidly eliminate the tubercle bacilli, leaving no evidence of exposure to TB; the tubercle bacilli can escape immune control and disseminate, causing so-called primary TB; or bacterial growth can be controlled but some bacilli survive and persist leading to latent tuberculosis infection (LTBI). The last two possibilities exist in a dynamic equilibrium, with episodes of disease and long periods of asymptomatic latent infection, or progressive and fatal disease both being possible outcomes.

An estimated 2 billion people worldwide have LTBI and may subsequently have reactivation of dormant bacilli, causing active disease many years after the infection [3]. This process is poorly understood, and only a few of the risk factors involved have been defined. Therefore detection and management of LTBI in persons who are at risk is very important. In developed countries, detection and management of LTBI is a key component of TB control [4,5]. In resource-constrained settings where testing for LTBI is not mandatory, the World Health Organization (WHO) recommends Isoniazid (INH) preventive treatment only for HIV-infected persons [6] and children < 5 years who are household contacts of persons with sputum smear-positive pulmonary TB [7]. However, in many cases, this leaves many MTB-infected people at risk, and there are therefore efforts to develop TB vaccines targeting the latently-infected population [8]. This is a long-term goal and in the meantime efforts are underway to identify those most at risk for targeted intervention.

For most of the past century, the only immunodiagnostic test for LTBI has been the tuberculin skin test (TST). Its major flaw is its inability to reliably distinguish individuals infected with *M. tuberculosis* from individuals sensitized to other mycobacteria, including BCG and most environmental mycobacteria [9]. A decade ago, interferon-γ release assays (IGRAs) were developed whereby interferon-γ titers were measured after *in-vitro* stimulation of peripheral blood mono-nuclear cells (PBMC) with antigens such as ESAT-6 and CFP-10 (immunodominant antigens expressed by members of the *M tuberculosis* complex, but not by most common mycobacteria and deleted in the BCG vaccine) [10]. Two commercial tools are currently available, and have been widely tested in many clinical situations and compared with TST, but most of these studies have been conducted in developed countries [11] with a low incidence of TB and restricted use of the BCG vaccine, which may compromise the specificity of TST since the antigens are contained in the tuberculin used for TST [9]. Therefore, the aim of this study was to evaluate the use of the most commonly-used IGRA, the QuantiFERON®-TB Gold In-Tube test (QFT-GIT), as compared with TST in the diagnosis of LTBI in Ethiopia, a country with a high burden of TB and routine BCG vaccination at birth.

## Methods

### Study participants and study sites

As an exploratory study, a formal power calculation was not performed, as TST screening is not routinely done in Ethiopia, and it was unclear what the expected response rate would be. However, it was known from prior work [12] that more than half of all healthy adults in the study region are responsive to ESAT-6 in a standard ELISA and a substantial proportion would therefore be expected to make some response in the QFT-GIT and TST. The study participants included were a total of 107 healthy male medical and paramedical students registered at the Faculty of Medicine, Addis Ababa University (AAU-MF), Ethiopia, volunteering to participate in a phase I TB vaccine trial conducted at the Armauer Hansen Research Institute (AHRI), Addis Ababa, Ethiopia (manuscript in preparation). Out of 110 participants from Addis Ababa University, Medical Faculty, who fulfilled all of the inclusion and none of the exclusion criteria, one participant did not return for TST reading and two did not undergo the TST because of withdrawal of their consent. A total of 107 students (Table 1) were thus included in the final analysis. All were male and the mean age was 20.9 (SD = 2.0) years. The mean body mass index (Kg/m^2^) was 18.9 (SD = 1.8). The study participants were enrolled in the study from December 2008 to February 2009. The general health status of all participants was assessed based on medical history, physical examination, chest radiography and blood tests including HIV. Exclusion criteria for this study were current or past history of chronic illness such as diabetes mellitus, hypertension, renal failure, hematological or other malignancies; clinical or radiological evidence of active tuberculosis; use of immune modulating drugs (steroids, immunosuppressive drugs or immunoglobulins) within the 3 months before enrollment in to the study; HIV seropositivity; and history of previous treatment for TB. The clinical laboratory tests, hematology, chemistry and HIV, were performed at the International Clinical Laboratories (ICL), Addis Ababa, Ethiopia. The QFT-GIT assay was carried out at the Armauer Hansen Research Institute (AHRI) laboratory, Ethiopia.

**Table 1:** Characteristics of study participants (n =107), students from Faculty of Medicine, Addis Ababa University, Ethiopia

**Characteristics**	**No(%)**
Department	
Laboratory Technology (LT)	50 (46.7)
Nursing	34 (31.8)
Others^1^	23 (8.4)
Year of study	
First year	43 (40.2)
Second year	47 (43.9)
Third year	17 (15.9)
Place of birth (Region)	
Addis Ababa	17 (15.9)
Amhara	30 (28.0)
Oromia	29 (27.1)
Southern Nations	22 (20.6)
Others^2^	9 (7.5)
Religion	
Christian	85 (68.2)
Muslim	22 (20.6)
Married	2 (1.9)
Claimed to have habit of Khat^3^ Consumption	9 (8.4)
Claimed to have habit of Cigarette Smoking	2 (1.9)

^1^ Midwifery, Radiography, Medicine, Dentistry and Pharmacy; ^2^ Tigray and Somali; ^3^Khat: *Catha edulis* is commonly known as Khat.

### Data collection and blood sample handling

A pretested questionnaire was used in order for the study physician to collect information on each participant’s health status and socio-behavioral factors. After taking medical history and performing physical examination, a blood sample was collected followed by the TST; and finally a Chest x-ray was taken. Blood was collected in QuantiFERON®-TB Gold test tubes (Cellestis Ltd, Australia*)* for QuantiFERON®-TB Gold In-Tube (IT) test; and BD EDTA tubes for HIV test and general laboratory test parameters (hematology and chemistry including blood glucose).

### Tuberculin skin test (TST)

Tuberculin (0.1 ml Tuberculin PPD RT 23; Statens Serum Institute, Copenhagen, Denmark) was injected intradermally on the ventral aspect of the left forearm. After 48-72 hours, the transverse diameter of the skin induration was measured with the ballpoint-pen method using a plastic ruler graduated in millimeters. A positive TST result was defined as an induration of ≥10mm [13].

### QuantiFERON®-TB gold in-tube (QFT-GIT)

The QuantiFERON®-TB Gold In-Tube (IT) test (Cellestis Ltd, Australia) was performed according to the manufacturer’s instructions. QuantiFERON®-TB Gold IT Analysis Software was used to analyze raw data and calculate results which were reported as Negative, Positive and Indeterminate [14].

### Statistical analyses

Comparisons were made using Fisher’s exact test, Student-*t* test or Mann–Whitney test where appropriate. Logistic regression was used to estimate crude and adjusted odds ratios (OR) to measure the effect of selected factors on the responses to TST and QFT-GIT tests. History of contact with TB patient was not included as a factor in the final analysis as there were only 4 out of the 107 study participants who identified a history of contact with a TB patient in their life time. Concordance between the results of the TST and QFT-GIT assay was assessed by using Kappa. According to recommendations regarding the interpretation of Kappa [15], values of 0.93-1.00 indicate excellent agreement whereas values of 0.81-0.92 and, 0.61-0.80 indicate very good and good agreement, respectively. Lower values indicate fair to slight, poor or no agreement. Correlation between the two LTBI diagnostic tests was assessed by spearman correlation. STATA statistical software version 8.0 (STATA Corporation, Texas, USA.) was used for data analysis. All p-values reported are based on two-tailed comparisons, with statistical significance set at p<0.05.

### Ethical considerations

The protocol of this study was approved by AAU-MF, Ethiopia and the Armauer Hansen Research Institute/All African Leprosy Rehabilitation and Training Center (AHRI/ALERT) Institutional Ethical Review Committees, Ethiopia. Approval was also obtained from the National Ethics Review Committee (NERC), Ethiopia. All of the study participants were provided with all relevant information about the study before they gave written consent.

## Results

### Tuberculin skin test (TST)

Fifty of the 107 participants (46.7%) had a positive TST result (TST≥10 mm). The mean age (SD) for study participants with positive and negative TST results was 21.02 (SD ± 1.63) and 20.40 (SD ± 1.21) years, respectively (P<0.05). Forty eight of the 107 study participants (44.9%, 95%CI: 35.2% to 54.8%) had BCG scar and of these 26 (54.1%, 95%CI: 39.2% to 68.6%) gave a positive TST result whereas of the 59 study participants who had no BCG scar, 24 (40.7%, 95%CI: 28.1% to 54.3%) gave a positive TST result (p>0.05). Employing a lower TST cut-off, 54/107 (50.5%, 95%CI: 40.6% to 60.3%) had an induration of >5mm, and at this cut off too, the impact of having a BCG scar was not statistically significant. The median size of induration for TST in participants with a BCG scar was 10 mm, with a range from 0 to 35 mm and for participants without BCG scar was 0 mm, with a range from 0 to 25 mm (p>0.05). There were no significant differences in TST positivity (assessed at TST≥10 mm) when the groups were analysed by the other variables listed in Table 2. However, in the logistic regression analysis (Table 2), the estimated odds of having a positive TST result (at a cut-off of ≥10 mm) was nearly 2 times higher (OR =1.6, 95% CI: 1.11 to 2.20; p = 0.01) as age increased by one year. Interestingly, habitual consumption of *Catha edulis* (a plant commonly known as Khat and the leaves of which are a mild stimulant) led to a 9 fold increase in risk (OR = 9.2, 95% CI: 1.41 to 59.69, p = 0.02).

**Table 2:** Results of logistic regression for a positive TST (TST≥10 mm) result

	**Bivariate analysis**		**Multivariable analysis**	
	**Crude OR**	**95% CI**	**Adjusted OR**	**95% CI**
Age	1.3	1.03-1.68	1.6	1.11-2.20
Nursing/LT	1.5	0.62-3.61	1.1	0.33-3.43
Others^1^/LT	2.0	0.72-5.30	1.0	0.22-4.81
Second year/First year	0.6	0.28-1.49	0.3	0.08-1.30
Third year/First year	1.1	0.35-3.31	0.5	0.07-3.18
Amhara/AA	0.9	0.26-2.84	1.2	0.24-5.65
Oromia/AA	1.4	0.42-4.60	1.4	0.31-5.92
Southern Nations/AA	0.1	0.01-1.38	0.1	0.01-1.63
Others^2^/AA	1.4	0.38-4.80	1.0	0.22-4.82
Muslim/Christian	1.2	0.46-3.01	0.7	0.19-2.83
Khat^3^ consumption (Yes/No)	2.5	0.58-10.38	9.2	1.41-59.69
BCG Scar (Yes/No)	1.7	0.80-3.72	1.5	0.57-3.82
BMI	1.0	0.79-1.20	0.7	0.54-1 .00

^1^Midwifery, Radiography, Medicine, Dentistry and Pharmacy; ^2^Tigray and Somali; ^3^Khat: *Catha edulis* is commonly known as Khat; *TST*: Tuberculin Skin Test; *LT*: Laboratory Technology; *AA*: Addis Ababa; *BMI*: Body Mass Index; *OR* = Odds Ratio; *CI*: Confidence Interval.

### QuantiFERON®-TB gold in-tube (QFT-GIT) assay

Forty-seven of the 107 participants (43.9%: 95%CI: 34.3% to 53.9%) had a positive result in the QFT-GIT assay. The mean age (SD) for study participants with positive and negative results was 21.04 (SD ± 1.72) and 20.42 (SD ± 1.15) years, respectively (p<0.05). Forty eight of 107 study participants (44.9%; 95%CI: 35.2% to 54.8%) had a BCG scar and of these 23 (47.9%; 95%CI: 33.3% to 62.8%) gave a positive QFT-GIT assay result whereas of the 59 study participants who lacked a BCG scar, 24 (40.7%, 95%CI: 28.1% to 54.3%) gave a positive result (p>0.05). There were no statistically significant differences in QFT-GIT assay positivity when analysed for any of the other variables listed in Table 3. However, in the logistic regression analysis, the estimated odds of having a positive QFT-GIT assay results was nearly 2 times higher (OR = 1.7, 95% CI 1.18-2.35; *p* =0.004) as age increased by one year, while Khat consumption was associated with a 10 fold increase in risk (OR =9.6, 95% CI 1.36-68.13, *p* = 0.02).

**Table 3:** Results of logistic regression for a positive QFT-GIT assay result

	**Bivariate analysis**		**Multivariate analysis**	
	**Crude OR**	**95% CI**	**Adjusted OR**	**95% CI**
Age	1.3	1.04-1.69	1.7	1.17-2.35
Nursing/LT	1.9	0.80-4.73	1.4	0.43-4.76
Others^1^/LT	2.5	0.92-6.94	2.1	0.43-10.10
Second year/First year	0.7	0.28-1.50	0.5	0.11-1.95
Third year/First year	0.9	0.30-2.87	0.8	0.11-5.43
Amhara/AA	1.1	0.33-3.65	1.5	0.30-7.62
Oromia/AA	1.3	0.40-4.47	1.2	0.28-5.60
Southern Nations/AA	0.2	0.02-1.77	0.2	0.02-2.34
others^2^/AA	1.7	0.48-6.16	1.1	0.22-5.42
Muslim/Christian	1.7	0.67-4.41	1.4	0.36-5.57
Khat^3^ consumption (Yes/No)	2.8	0.66-11.77	9.6	1.36-68.13
BCG Scar (Yes/No)	1.3	0.62-2.89	1.0	0.39-2.79
BMI	0.9	0.75-1.16	0.7	0.49-0.91

^1^Midwifery, Radiography, Medicine, Dentistry and Pharmacy; ^2^Tigray and Somali; ^3^Khat: *Catha edulis* is commonly known as Khat; *QFT-GIT*: QuantiFERON®-TB Gold In-Tube; *LT*: Laboratory Technology; *AA*: Addis Ababa; *BMI*: Body Mass Index; *OR* = Odds Ratio; *CI*: Confidence Interval.

### Agreement between TST and QFT-GIT assay in the diagnosis of latent TB infection

The overall strength of agreement between TST (≥10mm) and QFT-GIT assay was very good (Kappa = 0.83, *p* = 0.000), with concordant results in 98/107 (91.6%, 95%CI: 84.6% to 96.1%). Concordance between the two tests was good in participants with BCG scar (85.4%, Kappa = 0.71, *p* = 0.000), and excellent (96.6%, Kappa =0.93, *p* = 0.000) in those who lacked a BCG scar (Table 4). Out of the 107, 48 (45%) had a TST induration of 0mm and all were QFT-GIT negative.

**Table 4:** Agreement between TST (at two cut-offs) and QFT-GIT assay results stratified by BCG scar status

**Status of BCG scar**	**TST (mm)**	**QFT-GIT Pos**	**QFT-GIT Neg**	**Kappa**	***p *value**
BCG scar present	≥10	21	5	0.71	0.000
	<10	2	20		
BCG scar absent	≥10	23	1	0.93	0.000
	<10	1	34		
Total	≥10	44	6	0.83	0.000
	<10	3	54		
BCG scar present	>5	22	7	0.67	0.000
	<5	1	18		
BCG scar absent	>5	24	1	0.97	0.000
	<5	0	34		
Total	>5	46	8	0.83	0.000
	<5	1	52		

*TST*: Tuberculin Skin Test; *QFT-GIT*: QuantiFERON®-TB Gold In-Tube; *Pos*: Positive; *Neg*: Negative.

At a TST cut-off of >5mm, the overall strength of agreement between TST and QFT-GIT was also very good (Kappa = 0.83, *p* = 0.000), with concordant results in 98/107 (91.6%). Concordance between the two tests was also good in participants with BCG scar (83.3%, Kappa = 0.67, *p* = 0.000), and was excellent (98.3%, Kappa = 0.97, *p* = 0.000) in those who had no BCG scar (Table 4).

By taking the QFT-GIT assay as the gold standard for the diagnosis of latent TB infection (because of the expected higher specificity), we also looked at TST reactivity in those judged to be LTBI negative. TST positivity at a ≥10mm cut-off was not significantly different among QFT negative participants with or without a BCG scar. However, at a TST cut-off of >5mm, among participants with QFT-GIT negative results, 7 out of 25 (28.0%, 95%CI: 12.1% to 49.4%) participants with BCG scar were TST positive as compared to 1 out of 35 (2.9%, 0.07% to 14.9%) participants with no BCG scar (*p* =0.007).

**Figure 1:**
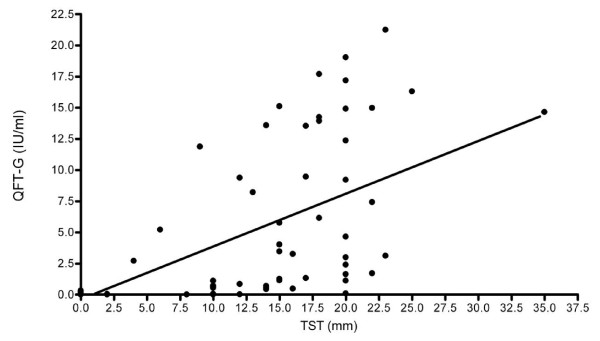
**Scatter plot of TST measurement and QFT-GIT assay (IU/ml) results.** Tuberculin skin test (TST) was performed by injecting 0.1 ml Tuberculin PPD RT 23 intradermally on the ventral aspect of the left forearm. After 48-72 hours, the transverse diameter of the skin induration was measured using a plastic ruler graduated in millimeters. A positive TST result was defined as an induration of ≥10mm. QuantiFERON®-TB Gold In-Tube (QFT-GIT) assay was performed according to the manufacturer’s instructions. The QFT-GIT (IU/ml) raw data was analyzed by using QuantiFERON®-TB Gold IT Analysis Software and reported as Negative, Positive and Indeterminate. In this figure, the correlation between the two latent tuberculosis infection (LTBI) test results, TST in mm and QFT-GIT in IU/ml, was assessed by spearman correlation; Spearman correlation coefficient = 0.81, *p<0.05.*

In addition, the correlation between TST (mm) and QFT-GIT assay (IU/ml) was assessed and found to be significant (spearman correlation coefficient = 0.81, *p<0.05*) as shown in Figure 1.

## Discussion

In this study we evaluated the use of the TST and QFT-GIT assays in the diagnosis of LTBI in a country with high burden of TB and routine BCG vaccination at birth. We observed strong agreement between TST (at a cut-off of ≥10mm) and the QFT-GIT assay. Our observation is comparable with a study conducted in a cohort of health care workers in India [16]. However, a recent community based study conducted in the Afar region of Ethiopia by Legesse *et al.*[17] reported weak agreement between the two tests.

In our study, all of the study participants were males, unlike the study by Legesse *et al.,* which reported a higher prevalence estimate in LTBI in males than females using TST, but no difference using the QFT assay. Despite this, the report by Legesse *et al.* found a lower rate of TST positivity (36.8%) than that we report here (46.7%). In addition, although 48% of the participants in the study by Legesse *et al.* had a TST induration of 0 mm, compared with 45% in our study, 42.7% of those were QFT-GIT positive, compared with 0% in our study. It therefore appears that the participants in the Afar region study were more likely to have a weak or absent TST response, regardless of their QFT status. Factors associated with TST false-negative reactions may include, but are not limited to, malnutrition, parasitic infections, HIV/AIDS infection, overwhelming TB disease, or difficulties with the method of TST administration and interpretation of the reaction [13]. Any of these factors may be an issue, as the two populations studied in the two studies are quite different – relatively affluent, urban and healthy based on medical history, physical examination, chest radiography and blood tests including HIV, in this study, relatively deprived and rural with no in depth evaluation of their health in the Legesse *et al.* study. It is therefore possible that the discordance seen in the study of Legesse *et al.* may reflect an increase in false negative TST reactions which could contribute to an under-estimate of the incidence of LTBI in this population.

The strength of agreement between TST and QFT-GIT is slightly weaker in BCG vaccinated as compared with the group without BCG vaccination at both TST cut-offs (See Table 4). The simplest explanation is the presence of false positive TST results due to sensitization to the TST reagent, Purified Protein Derivative (PPD), which contains mixture of antigens that are common to the MTB complex*,* environmental non-tuberculous mycobacterium strain and BCG [9]. This interpretation is supported by the stronger effect seen in BCG-vaccinated, QFT-negative donors at the lower TST cutoff. Some studies have shown that BCG-vaccinated individuals are more likely to have positive TST results [18,19]. Interestingly, however, the effects (if any) in this study are small: we did not find a significant difference in TST positivity rate, at cut-offs of either TST≥10 mm or TST > 5 mm, in BCG-vaccinated and BCG-unvaccinated participants. This finding could suggest either BCG vaccine administered in infancy has had a minimal effect on false TST positivity in young adults, or that the high rate of TST positivity due to true LTBI resulting from the high incidence of active pulmonary TB in our study setting has over-ridden the effect of BCG.

The BCG vaccine is not expected to have an effect on the QFT-GIT assay since the antigens that are contained in the assay are not present in any BCG strains [20]. Consistent with this, we saw no difference in QFT-GIT assay positivity rate between the BCG vaccinated (47.9%) and the non-vaccinated groups (40.7%). This is also consistent with studies elsewhere [21,22], and this might imply that BCG vaccination at infancy provides limited protection against MTB infection of adults. In addition to its specificity, studies have shown that QFT-GIT assay is as sensitive as TST by correlating its results with the degree of exposure (duration and proximity) to a source patient and the likelihood of acquiring infection from that source [11,22]. Therefore, we used the QFT-GIT assay as the baseline definition for the diagnosis of LTBI. Using this approach we were able to see an apparent effect of BCG vaccination on the TST. Among QFT-GIT negatives (those thought not to have LTBI), the rate of TST positivity in participants with a BCG scar was 28.0% (7/25), significantly higher than those without a scar (1/35 or 2.9%), at cut-off of >5mm. At a cutoff of ≥10mm there was no significant difference. These observations suggest the effect of BCG vaccine administered in infancy on TST reactivity in adults is weak. It is detectable at a cut-off of >5mm, but is unlikely to lead to larger indurations (≥10mm), which are presumably indicative of LTBI. Our hypothesis is in agreement with that indicated by a meta-analysis of several studies on the effect of BCG vaccination on TST, which indicated little effect 15 years after vaccination, especially on TST tests using the higher cutoff [23]. A more recent meta-analysis has also shown that the effect of BCG received in infancy on TST is minimal 10 years after vaccination [24]. This study confirms those finding and adds the observation that in countries with a high incidence of TB, where exposure to infection is likely to be substantial, does not appear to lead to significant boosting of BCG-induced PPD sensitivity.

Moreover, we have found a good correlation between the amount of IFN-*γ* (IU/ml) in the QFT-GIT assay and the magnitude of TST induration (mm) (spearman correlation coefficient = 0.81 and *P*<0.0001), which is also consistent with the hypothesis that LTBI leads to larger TST induration (Figure 1). This has been suggested by others as well [25,26].

In highly endemic areas, increasing age is usually associated with an increase in the LTBI rate, assessed by immunodiagnosis [27,28] which is easily explained by cumulative exposure to MTB with time. However, a new finding is that in the multivariate analysis, both age increment and Khat (*Catha edulis*) consumption were both found to be risk factors for LTBI, based on both TST (TST≥10 mm) and QFT-GIT assay. Khat is usually chewed in groups, and this often involves sitting together in small crowded rooms for long periods of time, especially in urban settings. In addition, the chewed leaves are spat out, a process which is likely to efficiently generate aerosols. If there is one TB patient within the group the chance of infecting others is probably relatively high. One recent study conducted in Afar region of Ethiopia where Khat consumption is a common practice, has also described it as risk factor for the development and spread of pulmonary TB [29]. Although smoking has been associated with LTBI [30-32], the number of smokers in our study was very low (2 out of 107 study participants) and hence we do not expect it to confound the effect of Khat consumption even if both habits are often found in the same individuals. While the association found here is strong, the small number of participants who consumed Khat means this finding cannot be regarded as definitive. Further studies to confirm the role for Khat consumption as a risk factor for LTBI should be conducted as Khat chewing is becoming a common practice all over the country, and indeed, throughout the Horn of Africa.

## Conclusion

In conclusion, this study showed strong agreement between TST (TST≥10mm) and QFT-GIT assay in healthy young adults residing in Ethiopia, a high TB incidence country, in spite of the routine BCG vaccination at birth in this population. While there is a possibility of generating false TST positivity at cut-off of TST > 5mm as the result of BCG vaccine administered at infancy, it appears unlikely to affect the diagnosis of LTBI at larger indurations (TST≥10mm).

## Authors’ contributions

AFD designed the study, collected data, involved in data analysis/interpretation and drafted the manuscript. JH involved in study design, data collection, analysis and write-up. MA involved in study design, analysis and write-up. MZ involved in study design and write-up. AM involved in analysis and write-up. AB involved in data collection and write-up. MC involved in laboratory work and write-up. LY involved in study design and write-up. GM involved in data analysis/interpretation and write-up. PB involved in study design and write-up. TMD involved in study design, data interpretation and write-up. AH involved in study design, data analysis and write-up of the manuscript. AA involved in study design, data analysis/interpretation and write-up of the manuscript and critically revised the manuscript. All authors read and approved the final manuscript.

## Competing interests

The authors declare that they have no competing interests.
